# Effects of Temperature on Fluidity and Early Expansion Characteristics of Cement Asphalt Mortar

**DOI:** 10.3390/ma13071655

**Published:** 2020-04-03

**Authors:** Xiaohui Zeng, Huasheng Zhu, Xuli Lan, Haichuan Liu, H.A Umar, Youjun Xie, Guangcheng Long, Cong Ma

**Affiliations:** 1 School of Civil Engineering, Central South University, Changsha 410075, China; zxhzlh@126.com (X.Z.); lanxuli2020@csu.edu.cn (X.L.); 13086611297@163.com (H.L.); xieyj@mail.csu.edu.cn (Y.X.); scc2005@csu.edu.cn (G.L.); macgyh090@csu.edu.cn (C.M.); 2 Department of Civil Engineering, Ahmadu Bello University, Zaria 800001, Nigeria; haumar12@gmail.com

**Keywords:** CAM I, temperature, fluidity, expansion, pH value, demulsification

## Abstract

In order to solve the problems of the sudden loss of fluidity and low expansion rate of CAM I (cement asphalt mortar type I) in a construction site with high environmental temperature, this paper studies the effect of temperature on the fluidity, expansion ratio and pH value of CAM I. The mechanism of action was analyzed by IR (infrared spectrometry), SEM (scanning electron microscopy) and other test methods. The results showed that a high temperature accelerates aluminate formation in cement paste. Aluminate adsorbs emulsifiers leading to demulsification of emulsified asphalt, and wrapped on the surface of cement particles, this causes CAM I to lose its fluidity rapidly. The aluminum powder gasification reaction is inhibited, resulting in an abnormal change in the expansion ratio. Based on findings, the application of an appropriate amount of superplasticizers can effectively improve the workability and expansion characteristics of CAM I at a high temperature.

## 1. Introduction

**Figure 1:**
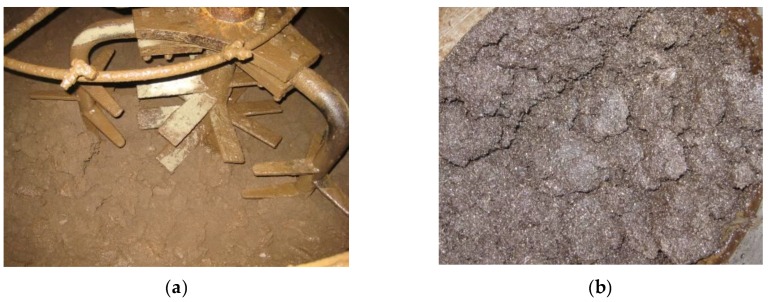
Flocculation and demulsification of emulsified asphalt in CAM I (**a**) Unable to mix, (**b**) Flocculation.

**Figure 2:**
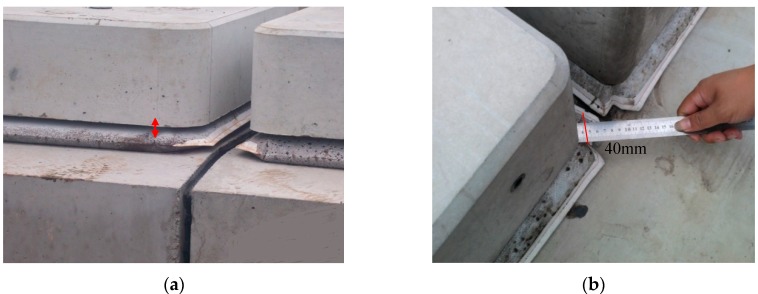
The gap between filling layer and track slab (**a**) vertical direction, (**b**) horizontal direction.

The ballastless slab track structure is an advanced track structure which is used widely in high speed railway in countries, such as Germany, Japan and China, due to its advantages of high stability, high durability and low maintenance compared to ballasted track structure. Nowadays, two main forms of ballastless slab track (CRTS I and CRTS II) are used in China, and although there are some differences in slab structures, the cement asphalt mortar (short for CAM I) layer is present in both slab tracks. CAM I is an interlayer injected in between the track and the bottom plate of the high-speed railway ballastless slab track. Its main components are: emulsified asphalt, Portland cement, fine aggregates, water, expansion agent, aluminum powder and other admixtures. CAM I used in CRTS I and CRTS II slab tracks is divided into CAM I and CAM II respectively. CAM I offers tremendous applications to the ballastless slab structure, including supports and leveling—which helps to adjust track precision; it also absorbs vibration and helps in vibration isolation. The thickness of the CAM I layer varies due to differences of slab track structure and the type of CAM I used [1,2,3,4,5].Studies showed that the environmental temperature has a serious impact on the quality of CAM I perfusion in a construction site. At high temperatures, the CAM I paste in the mixing will lose fluidity instantaneously, and causes the phenomenon of demulsification and flocculation (Figure 1). After completing the perfusion, insufficient expansion of CAM I results in gaps between the filling layer and the track slab (depth > 40 mm) (Figure 2). These problems have seriously delayed the construction progressions and threatened the safety of high-speed trains, so need to be resolved.

Some studies have been conducted on the effects of temperature on the performance of CAM. The temperature sensitivity and mechanical properties of CAM are closely related to the thermal dependence of emulsified asphalt, and the compressive strength of CAM decreases with increasing curing temperature [6,7]. Wang et al. put forward a temperature stability coefficient (TSC) that effectively characterizes the temperature dependence of the strength of CAM [8]. Wang et al. [9,10] and Kong et al. [11] studied the effect of test temperature (0–40 °C) on the compressive strength of CAM, and based on the experimental observations, the empirical equations of compressive strength and temperature of CAM were obtained. Hu et al. used a concrete pressure bleeding instrument to study the compressive strength of CAM in water at different temperatures (20, 40, 60 °C) and pressure (0–0.5 MPa), and found that the compressive strength of CAM will decrease with increasing water temperature [12]. Temperature also has an important influence on the stress relaxation process of CAM. As temperature decreases, the stress relaxation rate and stress relaxation modulus of CAM increase gradually [13].

Zhang et al. [14] studied the effect of temperature (0, 20, 40 °C) on the rheological properties of fresh cement asphalt paste using Brookfield DV-III + ULTRA rheometer; it was found that the yield stress of cement asphalt increased with increasing temperature, and cement asphalt mixed with cationic emulsified asphalt is more sensitive to temperature than anionic emulsified asphalt; they believed that the adsorption of emulsified asphalt by cement particles may be the cause of these phenomena, but the reason for this adsorption is not explained clearly. At higher temperatures, the adsorption behavior of cement particles and emulsified asphalt in fresh cement asphalt paste is more prominent, resulting in increased viscosity and reduced workable time, but the microscopic mechanism is not clear [15]. Therefore, it is necessary to explore the temperature-sensitive micro-mechanisms, such as the working time and expansion rate of fresh CAM paste.

To that end, this paper tests the change of CAM I fluidity, expansion ratio and pH value with time at different temperatures by simulating on-site construction conditions. The stability and evolution mechanism of the CAM I system at different temperatures were studied, and an effective solution to the above construction problems was explored. The purpose was to optimally mix the proportion of CAM I and provide guidance for construction quality control of CAM I.

## 2. Experiments

### 2.1. Materials

In order to save mixing time and ensure the stability of CAM I, cement, sand, aluminum powder and other solid admixtures were premixed and made into dry materials, while liquid admixtures were added to emulsified asphalt. Field construction products included dry materials and emulsified asphalt. Properties of CAM-I dry materials (Table 1) and emulsified asphalt (Table 2) meet the requirements of Chinese code [16] (“Provisional technical conditions for cement asphalt mortar and resin pours for CRTS I slab ballastless track of Passenger Dedicated Railway”). Table 3 shows the mix proportions of CAM I.

Pouring bag: The pouring bag is made of polyester non-woven fabric with a mass per unit area of 105 g/m^2^. Other performance indicators meet the requirements of Chinese code [16].

Water: tap water is used throughout the experiments.

### 2.2. Testing Methods

#### 2.2.1. Test of Fluidity

**Figure 3:**
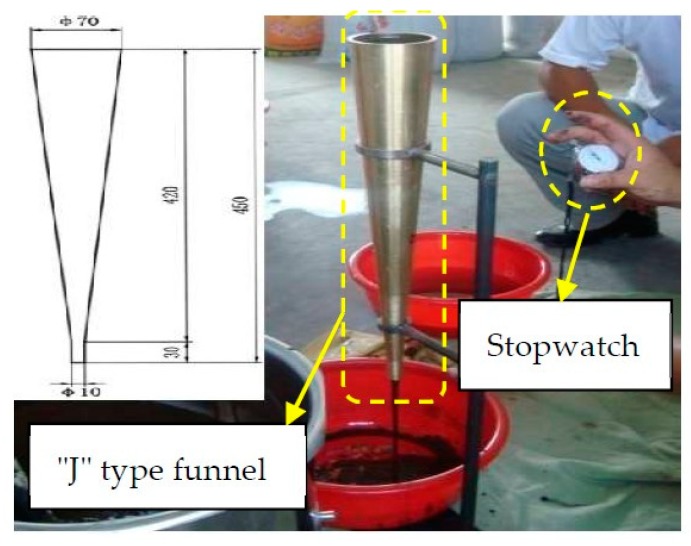
Testing device of the fluidity of CAM I.

According to Chinese code, the fluidity of CAM I was measured using a "J" type funnel (Figure 3). The testing device includes a funnel (made of brass), a bracket (made of iron) and a stopwatch (accuracy 0.1 s). The test steps are as follows: 

(1) The funnel is vertically erected on the bracket.

(2) The sample is injected into the funnel; the right amount of sample is discharged from the outlet; and the outlet is pressed by fingers so that the sample fills the funnel and the surface is leveled.

(3) Release your finger and the mortar flows out. Use a stopwatch to measure the time it takes for the mortar to flow continuously from the beginning to the end, which is the fluidity of the sample—t (in seconds).

(4) Carry out a fluidity test on the same sample every 10 min and draw a fluidity curve (Figure 6); that is, the correspondence between the fluidity and the accumulated time.

(5) Each group of samples is tested three times for fluidity and working time, and the mean value is taken.

#### 2.2.2. Test of the Early Expansion Ratio

**Figure 4:**
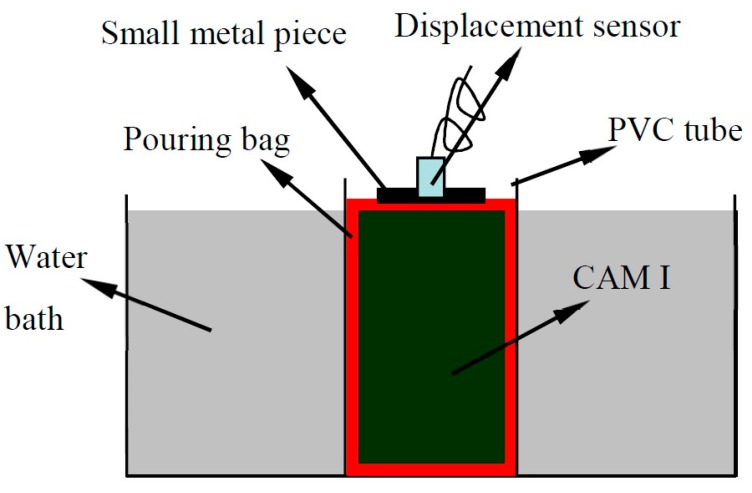
Testing device of the early expansion ratio of CAM I.

We measured the early expansion ratio of CAM I with a self-designed device (Figure 4); this device can continuously monitor the expansion ratio of pastes by a displacement sensor. The temperature of a sample is adjusted by heating in a water bath. The test steps are as follows:

1 The fresh CAM I paste with specified temperature is injected into the φ 10 × 20 cm pouring bag, tied and put it into the φ 10 cm PVC tube.

2 Press the sample by lighter metal sheet; then the PVC tube is placed in a water bath with a specified temperature, and a displacement sensor is placed on the metal sheet.

3 Set to record a displacement value every 5 min; each group of samples is tested three times, and the mean value is taken.

#### 2.2.3. Test of pH Value

The pH value of sample is measured by a pH meter (Delta320); each sample is tested three times, and the mean value is taken.

#### 2.2.4. Microstructural Analysis

**Figure 5:**
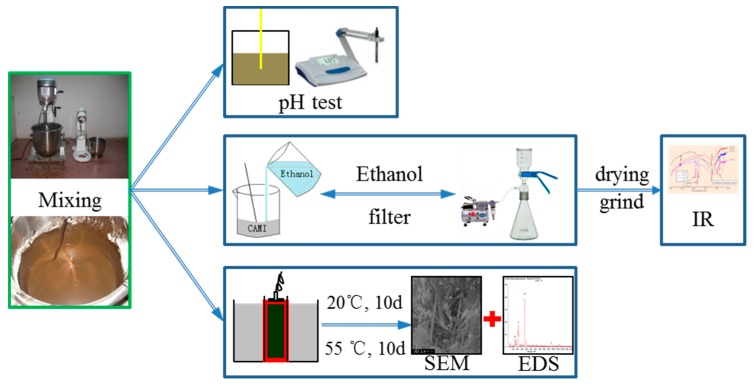
The diagram of experimental steps.

1 Infrared spectrometry (IR): The paste hydration for 5 min is stopped hydration by ethanol, and then IR analysis was carried out (Process the sample as shown in Figure 5).

2. Scanning electron microscopy (SEM): The samples of CAM I (curing at 20 °C and 55 °C) are analyzed with scanning electron microscopy (SEM) and a X-ray energy dispersive spectrometer (EDS) at the age of 10 days. The experimental steps are shown in Figure 5.

As summarized in Table 4, the experiment of this study was divided into three parts: one was the experiment of the influence of temperature on the fluidity and expansion rate of CAM I paste (numbers 1 and 2); the other was the experiment of exploring the micro mechanism (numbers 3, 4, 5, 6, 9 and 10); and the last was the experiment of solving the problem (numbers 7 and 8). Thus, the experiments were not performed at same temperature. Since CAM I paste has completely lost its fluidity at 55 °C, the expansion rate cannot be tested at 55 °C. When testing the variation of pH value of CAM I paste with durations at different temperatures, it was found that the pH value of CAM I paste changed most abnormally at 55 °C compared to 20 °C. Therefore, the changes of pH value for each system of CAM I paste with time at both 20 °C and 55 °C were tested to explore the direct cause of the instability of pH value of CAM I paste at high temperature. When performing microstructural analysis, considering that the precision of an IR test is higher than that of SEM in the analysis of phase composition, only two samples at 20 and 55 °C were analyzed by SEM, while all samples at four temperatures were analyzed by IR. Due to the daily maximum temperature below 40 °C during construction, only the effects of superplasticizer on the fluidity and expansion rate of CAM I paste were studied at 45 °C.

## 3. Results and Discussion

### 3.1. Analysis of Fluidity and Expansion Ratio of CAM I 

**Figure 6:**
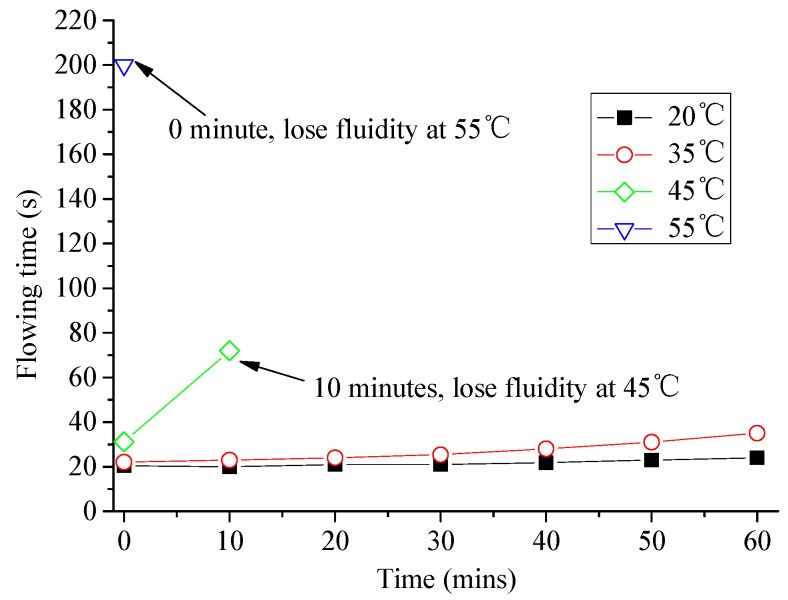
Effects of temperature on fluidity of CAM I paste.

Effects of temperature on fluidity of CAM I paste are shown in Figure 6. In the process of mixing, at 55 °C, the paste lost its fluidity and became flocculent immediately. At 45 °C, the paste also thickened and lost its fluidity about 10 min after mixing. The fluidity of paste at 20 °C and 35 °C increases slowly with time. The fluidity of paste at 20 °C remains in the range of 26 s after 1 h, and the fluidity of paste at 35 °C was found to maintain at 18~26 s within 30 min.

**Figure 7:**
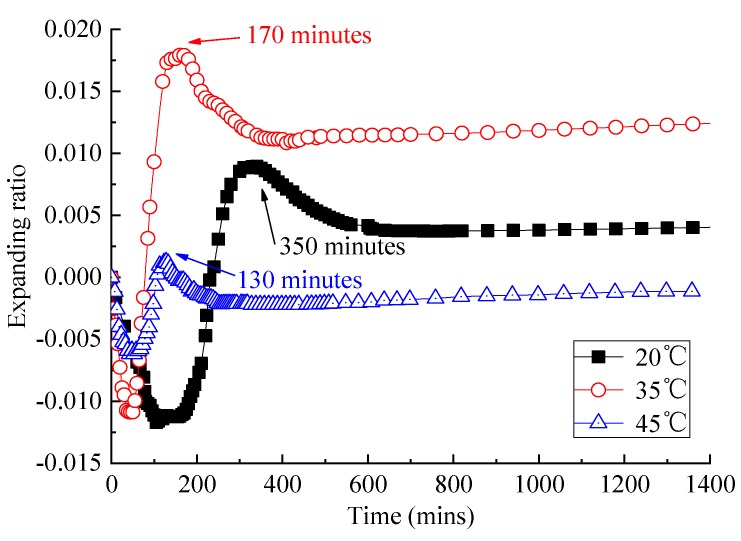
Expansion ratio of the CAM-I changes with time at different temperature.

As is shown in Figure 7, with the increase in temperature, the beginning time and the end time of expansion of CAM I paste are advanced. When the temperature was at 20 °C, the paste began expanding at about 150 min and stopped at about 350 min. When the temperature was at 35 °C, it begun expanding at about 45 min and stopped at 170 min. When the temperature was at 45 °C, it started and stopped early, at approximately 50 min and 130 min. This may be related to the fact that the rise of temperature can accelerate cement hydration.

When the temperature rises from 20 to 35 °C, the expansion ratio of CAM I paste increases significantly, and the final expansion ratio increases more than twice. When the temperature rises from 35 to 45 °C, the final expansion ratio of CAM I paste is decreased by nearly 70%, and the volume even shrinks in the end. In alkaline conditions induced by cement hydration, the aluminum powder reacts with OH^−^ produce H_2_ in the solution, resulting in a decreased pH value of the solution. This indicated that the expansion ratio is closely related to cement hydration. Chemical reactions were as followed [17]:

(1) $$\begin{array}{l} 2Al+3Ca^{2+}+6H_{2}O+6OH^{-}=3CaO\cdot Al_{2}O_{3}\cdot 6H_{2}O+3H_{2}↑\\ 2Al+Ca^{2+}+2OH^{-}+6H_{2}O=C_{a}[Al(OH{)}_{4}{]}_{2}+3H_{2}↑ \end{array}$$

### 3.2. Analysis of pH Value on Different Systems of CAM-I Paste

The pH value is a momentous parameter of cement hydration. In order to find out the reason for the abnormality of fluidity and expansion ratio of CAM-I at high temperature, the changes of pH for each system of CAM I paste at different temperatures were tested. 

#### 3.2.1. Change of pH Value of Emulsified Asphalt–Sand System 

**Figure 8:**
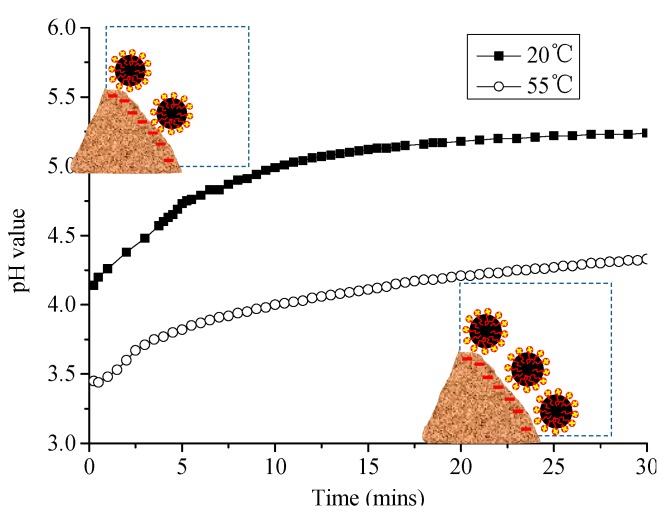
pH value of emulsified asphalts–sand system changes with time at different temperatures.

As shown in Figure 8, the pH value of slurry gradually increases during the stationary time and increases very slowly after 15 min in the emulsified asphalt-sand system. Emulsified asphalt used in this study is cationic emulsified asphalt. The quaternary ammonium salt N^+^ cation formed by dissociation was adsorbed on the surface of sand, so that the electrical double layer on the surface of emulsified asphalt particle was compressed, and the concentration of OH^−^ in the solution increased. During the whole process, the pH value was still smaller than 7.0, meaning the slurry was still acidic. With the increase of temperature, the degree of ionization of electrolyte increases in the system, and the concentration of OH^−^ in the solution increases, so the pH value decreases. Since the emulsified asphalt is an acid and alkali resistant material, the slurry can still be stable, which can also be seen from the fact that the consistency of the slurry increases with time and does not change significantly over time.

#### 3.2.2. Change of pH Value of Emulsified Asphalt–Water System 

**Figure 9:**
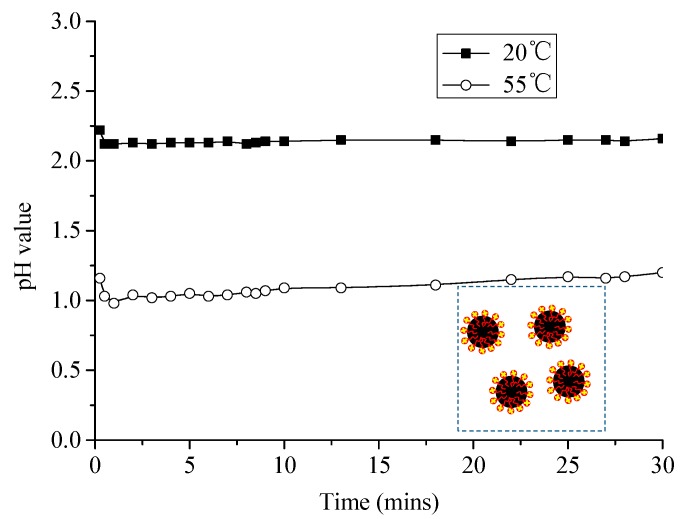
pH value of emulsified asphalt–water system changes with time at different temperatures.

As shown in Figure 9, the pH of the slurry almost did not change with time in the emulsified asphalt–water system (except for the pH value of slurry at 0.25 min, which was too high due to the initial contact), which indicates that the emulsified asphalt–water system is a relatively stable system. The pH value of the slurry decreases from 2.0 to 1.0 when the temperature increases from 20 to 55 °C. This indicates that the concentration of H^+^ increases by 10 times; this is because the high temperature accelerates the ionization of electrolyte.

#### 3.2.3. Change of pH Value of Emulsified Asphalt–Cement System 

**Figure 10:**
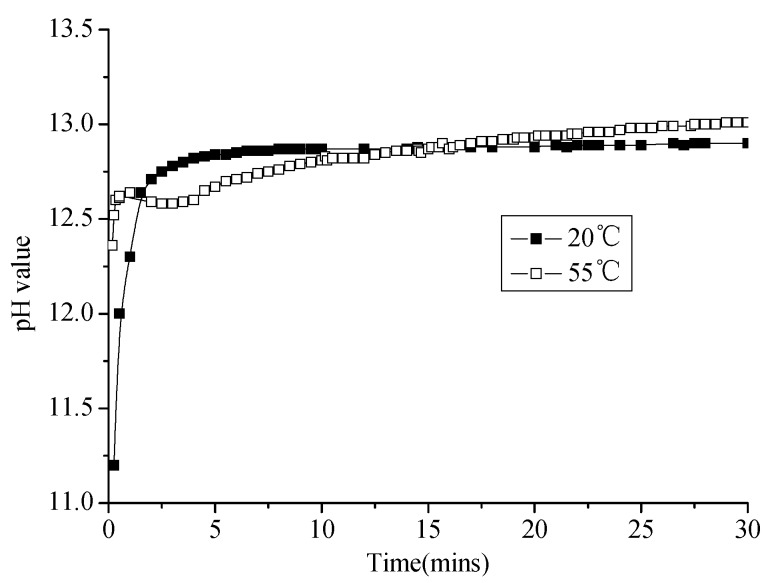
pH value of emulsified asphalt–cement system changes with time at different temperatures.

Figure 10 shows the change of pH value for the emulsified asphalt–cement system over time. Obviously the pH value of emulsified asphalt–cement system changes with both time and temperature. At 20 °C, the pH value of the slurry increases constantly with time; the pH value of the slurry firstly increases, then decreases and finally increases with time at 55 °C. The initial pH value was above 12.0 at 55 °C. At the same time, the consistency of the slurry significantly increases with time at 55 °C in the mixing process. This result indicated the rapid loss of fluidity and the abnormal change of expansion ratio of CAM I paste at high temperature may be attributed to the instability of emulsified asphalt–cement system at high temperature.

At high temperature, one of the possible reasons responsible for the instability of the emulsified asphalt–cement system is the high pH value. Because the emulsified asphalt used in CAM-I is cationic emulsified asphalt, in order to increase its stability, the pH of the slurry must be adjusted to a value that is less than 7.0. However, when emulsified asphalt and cement are mixed by a mixer, the initial pH value of slurry reaches above 12.0 instantly. Therefore, the cement particles may be wrapped by the emulsified asphalt that has been demulsified, which delays the hydration of cement. At 55 °C, we adjusted the pH value of the emulsified asphalt to 13.0 with NaOH solution, but the color of emulsified asphalt was still brown, and it has great fluidity, and there is no obvious demulsification. We could draw conclusions that, on one hand, the emulsified asphalt has a great resistance to high temperature, acid and alkali; on the other hand, demulsification is not caused by a high pH value.

#### 3.2.4. Change of pH Value of CAM I Paste 

**Figure 11:**
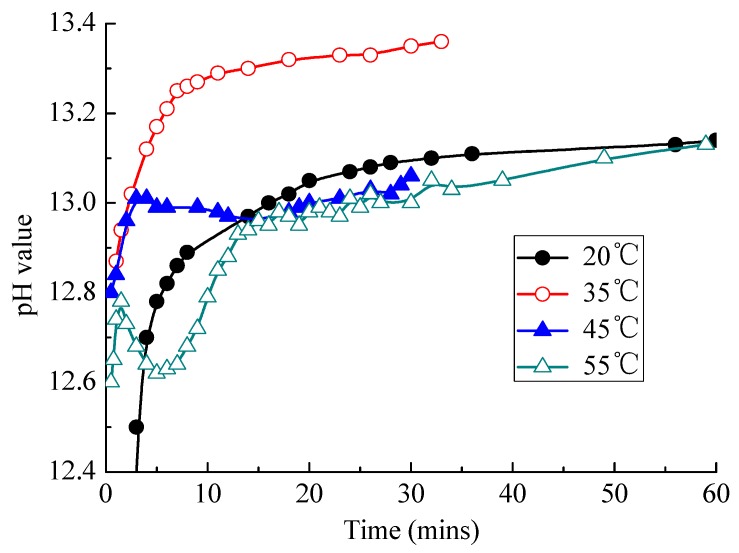
Effects of temperature on changes of pH value of CAM-I with time.

The pH value of CAM-I paste at different temperatures was tested in Figure 11.When CAM-I is mixing with water, the pH value of paste reaches a high level in a moment and increases with time, which may be attributed to the large amount of Ca(OH)_2_ produced by early hydration of cement. High temperature can promote ionization of various ions and accelerate cement hydration. The pH value of paste also rises when temperature increases from 20 to 35 °C. Since the reaction of aluminum powder requires a certain concentration of OH^−^, the rise in temperature accelerates the expansion caused by the aluminum powder gas-generating reaction.

However, when the temperature was at 45 or 55 °C, the pH value of slurry behaved abnormally with an increase in hydration time. The pH value of paste decreases first, and then increases with time; moreover, the duration of the rising and falling period becomes shorter with the increase of temperature. When the temperature was 45 °C, the rising period was about 5 min, and the falling period was about 10 min. When temperature rises to 55 °C, the rising period lasts only 1 min, while the falling period lasts only 4 min. All these phenomena indicated that a mutation occurs in the system at high temperature, and this mutation caused an abnormal change in the pH value of paste over time, which in turn caused an abnormal expansion due to the aluminum powder gas-generating reaction r.

There are mainly three reasons for the reduction of pH value at high temperature. 

One reason was that the crystallization of Ca(OH)_2_ consumes some OH^−^. High temperature greatly accelerates the dissolution rate of cement minerals, so that the concentration of Ca(OH)_2_ in the solution increases rapidly. However, since the solubility of Ca(OH)_2_ decreases when temperature increases, Ca(OH)_2_ in the solution becomes saturated promptly; then it crystallizes and precipitates. Consequently, the inducted stage of cement hydration is shortened, and the accelerated stage of cement hydration quickly begins. 

Another reason was that the rapid precipitation of aluminate hydrates such as AFt results in the decrease of pH value. As shown in Equations (2) and (3), 4 mol OH^−^ should be consumed if 1 mol AFt or aluminate hydrates is produced; as a result, the pH value of paste become lower.

(2) $$6Ca^{2+}+2Al{\left(OH\right)}_{4}{}^{-}+3SO_{4}{}^{2-}+4OH^{-}\to C_{6}A{\bar{S}}_{3}H_{32}$$

(3) $$4Ca^{2+}+2Al{\left(OH\right)}_{4}{}^{-}+2X^{-}+4OH^{-}\to C_{4}AX_{2}H_{n}$$

(4) $$C_{3}A+CH+12H\to C_{4}AH_{13}$$

The reaction of Equation (4) generally takes place at a high concentration of Ca(OH)_2_. C_4_AH_13_ is a hexagonal flake hydrate, which usually turns into AFt promptly; however, a high concentration of Ca(OH)_2_ will stabilize it [18], and obviously, a high temperature makes this possibility greatly increase. The reaction of Equation (4) usually results in the flash setting of cement.

The third reason was that high temperature accelerates cement hydration, and a series of physical and chemical effects lead to the emulsified asphalt to flocculate, demulsify and form floccule (visible to eyes). The floccule adsorbs a large amount of water and ions (a massive amount of water exudes from the floccule when squeezed by hand), and cement particles are wrapped by asphalt which prevents further dissolution of cement minerals, thereby causing decrease in pH value of paste.

### 3.3. Analysis of Hydration Phase in CAM-I

**Figure 12:**
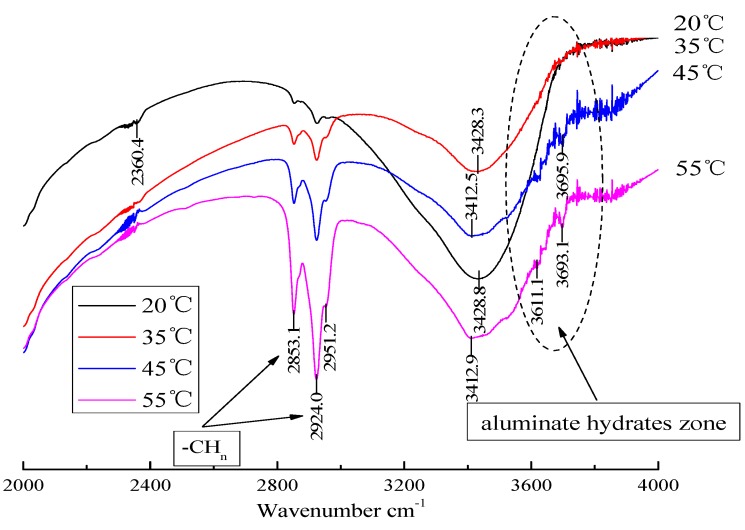
IR analysis of CAM-I hydrates at different temperatures (hydration for 5 min).

It can be seen from the above analysis that some mutation may occur in the system at a high temperature, which causes the flocculation and demulsification of emulsified asphalt and the abnormal change of pH value and expansion rate of CAM-I paste. In order to clarify the micro mechanism, the CAM-I samples (20, 35, 45 and 55 °C) hydrating for 5 min were analyzed by infrared spectrum (IR), as shown in Figure 12. Clearly, 2853.1 cm^−1^ is the asymmetric stretching vibration peak of −CH_3_; 2924.0 cm^−1^ is the symmetric stretching vibration peak of −CH_2_; 2360.4 cm^−1^ is the stretching vibration peak of quaternary ammonium salt N^+^; both alkyl and quaternary ammonium groups are associated with emulsifiers of emulsified asphalt. The peak around 3400 cm^−1^ is related to the stretching vibration peak of −OH (cement hydrates mostly contain −OH groups; among them, the Tobermorite is 3440–3460cm^−1^; hard xonotlite is 3420–3460cm^−1^; AFt is 3420 cm^−1^; AFm is 3480 cm^−1^; hydrated calcium sulphate is 3400 cm^−1^). The peak of 3600–3700 cm^−1^ is mainly related to aluminate hydrates [19].

Figure 12 showed that both the asymmetric stretching vibration peak of −CH_3_ and the symmetric stretching vibration peak of −CH_2_ increase significantly when the temperature increases; that is, the content of emulsifier-related substance increased in the sample. It showed that the emulsifier adsorbed on the surface of asphalt particles is dispersed in the paste (i.e., emulsified asphalt may demulsify). In addition, a peak associated with the aluminate gel appeared in the 45 and 55 °C samples, which indicates that high temperature promotes the reaction associated with the formation of aluminate hydrates [20]. 

**Figure 13:**
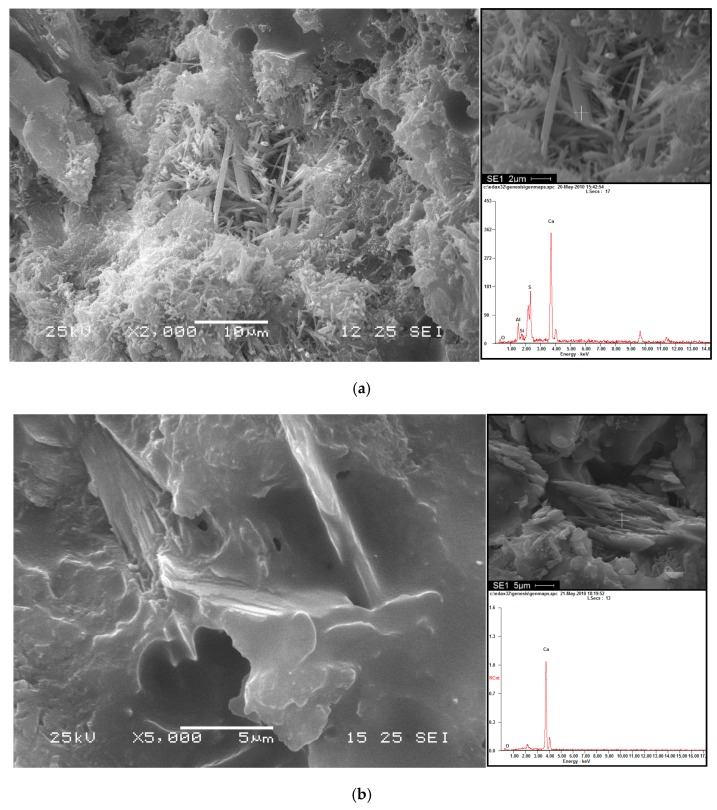
SEM and EDS analysis of CAM-I hydrated in different temperature (**a**) 20 °C, (**b**) 55 °C (hydration for 10 days).

The CAM-I samples (curing temperatures of 20 and 55 °C) were analyzed by scanning electron microscopy (SEM) and energy dispersive spectrometry (EDS) at the curing age of 10 days. According to the SEM and EDS photographs of the sample cured at 20 °C (Figure 13a), many loose needle shaped hydration products were observed, and the EDS analysis of the spicules indicated that their main elements were Ca, S and Al, so the needle-shaped hydration products were ettringite (AFt) [19]. In the SEM and EDS analysis of CAM-I samples cured at 55 °C (Figure 13b), the cement hydration products cannot be seen clearly except for Ca(OH), and the asphalt film was clearly observed (Fu [13], Tian [21] and Tyler [22] observed this microstructure in their studies). Therefore, a high temperature accelerated the demulsification of emulsified asphalt in the paste.

The emulsifier used in the emulsified asphalt is similar to the traditional wood calcium and naphthalene-based water reducer, which were both ionic surfactants adsorbed on the surface of the asphalt particles, making them bring positive charges. The stability of the emulsified asphalt was mainly related to the adsorption strength of the emulsifier on the surface of asphalt particles and the concentration of the emulsifier in the solution. The hydrolysis reaction of C_3_A after adding water at high temperature is as follows [20]:

(5) $$C_{3}A\to 3Ca^{2+}+2Al{\left(OH\right)}_{4}{}^{-}+4OH^{-}$$

$Al{\left(OH\right)}_{4}^{-}$ can be regarded as aluminum hydroxide gel with OH^−^ adsorbed onto it. Corstanje et al. [23] proposed that there is amorphous Al(OH)_3_ formed on the surface of C_3_A. Skalny [24] found that an aluminum-rich layer exists on the surface of the C_3_A particles and retards the hydration of C_3_A. Barnes [20] considered the aluminum-rich layer to be a coprecipitate of Ca(OH)_2_ and Al(OH)_3_ or only Al(OH)_3_. Zhang et al. [14] found in studies that the adsorption amount of emulsified asphalt by cement particles increased at high temperatures. Additionally, from the analysis results of IR, it can be seen that the emulsifier content in the paste increased at high temperatures. Therefore, z high temperature accelerated the hydration of C_3_A, and produced a negatively charged aluminate hydrate to adsorb emulsifiers and emulsified asphalt particles. The emulsifier adsorption layer on the surface of the asphalt particles became loose, the electric double layer on the surface of the particle was changed [25] and the electrostatic repulsion was weakened, which resulted in the asphalt particles gradually aggregating, flocculating and then demulsifying. (The macro performance is the sudden loss of fluidity of CAM-I paste and the abnormality of expansion rate.) The emulsified asphalt demulsified to form a film, and adsorbed a large number of ions, which were wrapped on the surfaces of cement particles to prevent further dissolution of cement clinker and diffusion of water [13,21]. On one hand, this reduced the concentration of OH^−^ in cement paste, thereby lowering the pH value. On the other hand, at high temperature, the absorption peak of emulsifier and aluminate appeared simultaneously in the IR spectrum of the sample hydrated for 5 min. The cement hydration rate was also slowed down so that the cement hydration products could not be seen in the sample of 55 °C at the 10 day mark except for Ca(OH)_2_. Moreover, when EDS analysis was performed on the surface, the peaks of elements other than Ca were very weak. Figure 16a presents a schematic illustration of the demulsification of emulsified asphalt at a high temperature.

### 3.4. Effect of Superplasticizer on the Fluidity and Expansion Rate of CAM-I

From the above analysis, it can be seen that the high temperature accelerated the hydration of C_3_A and generated more aluminate hydrates which adsorbed emulsifiers, leading to the demulsification of the emulsified asphalt, and the paste lost its fluidity.

**Figure 14:**
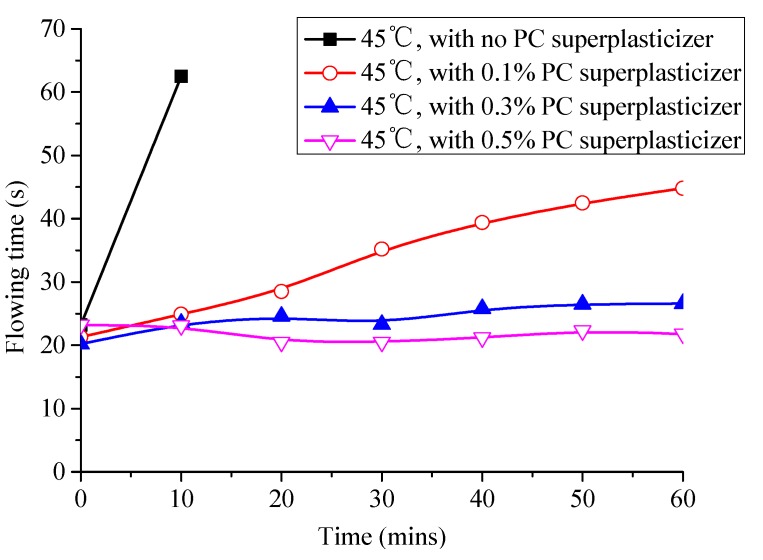
Effect of superplasticizer on the fluidity of CAM I paste.

**Figure 15:**
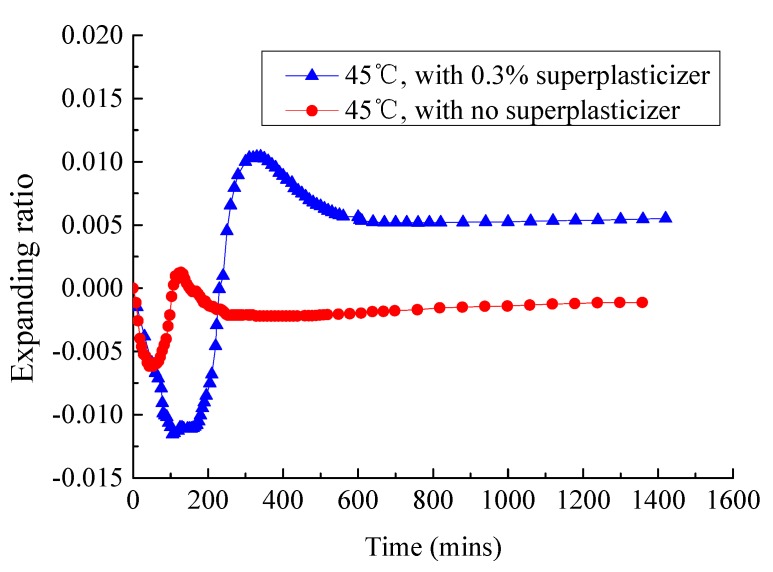
Effect of superplasticizer on the expansion ratio of CAM I paste.

Therefore, the best way to solve the problem of sudden loss of fluidity and abnormal change of expansion rate is to weaken the adsorption capacity of aluminate hydrates toward emulsifiers and asphalt particles. In this study, a certain number of superplasticizers (percentage of cementitious material mass) are added to CAM-I paste to study its effect on the working time and expansion ratio of paste based on competitive adsorption principle [26,27,28]. As shown in Figure 14, the fluidity of paste without superplasticizer increases from 23.2 s to 62.5 s after 10 min when the temperature is 45 °C, and the paste loses fluidity. However, the paste can maintain high fluidity within 60 min and can be used for construction after adding 0.1% superplasticizer. The fluidity of paste can maintain for 18–26 s within 60 min when the amount of superplasticizer is increased to 0.3%. The fluidity of paste when the amount of superplasticizer was 0.5% was lower than that of 0.3% superplasticizer during 0–10 min; the phenomenon was also found in previous studies [29], which was interpreted as “aftereffect of superplasticizer”. As shown in Figure 15, the volume of paste without superplasticizer eventually shrunk, but the expansion ratio of paste was greatly improved by 0.55% when the amount of superplasticizer was 0.3%.

**Figure 16:**
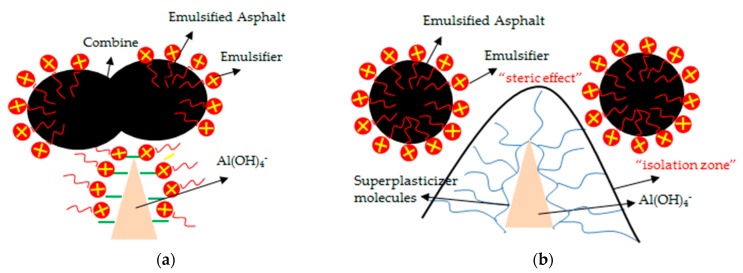
Schematic illustration of competitive adsorption of superplasticizer molecules and asphalt particles (**a**) Without superplasticizer, (**b**) With superplasticizer.

At a high temperature (about 45 °C), the emulsifier was adsorbed by the aluminate hydrates formed in the early stage of cement hydration, resulting in the system instability. The emulsified asphalt demulsified and formed a film on the surface of cement particles, which will prevent further cement hydration (Figure 13b). The superplasticizer molecules will be adsorbed on the surfaces of aluminate hydrates [30]. Many long molecular chains form an "isolation zone" between emulsifiers and cement minerals that hinders the adsorption of emulsifiers (Figure 16b). Therefore, the emulsified asphalt remains stable and the cement particles could be further hydrated. The fluidity and expansion ratio of CAM-I paste was improved.

## 4. Conclusions 

Based on the results of this experiment and the discussions above, the following conclusions can be drawn.

(1) When the temperature was 20–35 °C, the fluidity of CAM I paste increased slowly with time, but the paste became thickened and flocculated rapidly when the temperature was above 45 °C.

(2) When temperature increased from 20 °C to 35 °C, the expansion ratio of CAM-I paste increased with the increase in temperature, but the expansion ratio of CAM-I decreased with the increase in temperature when the temperature was above 45 °C.

(3) High temperatures accelerate the formation of aluminate hydrates in cement paste. The adsorbed emulsifier leads to demulsification of emulsified asphalt; then asphalt film wraps the surface of cement particles, which causes CAM-I paste to lose fluidity quickly, and the gas generating reaction caused by aluminum powder is inhibited, resulting in abnormal change of pH value and decrease of expansion ratio in the paste.

(4) The superplasticizer can stabilize the emulsified asphalt by forming an "isolation zone" between the emulsifier and the cement particles, which can improve the working time and expansion ratio of the CAM-I paste.

## Figures and Tables

**Table 1:** Properties of CAM-I dry materials.

Aluminum Powder	Portland Cement	Sand	24 h Volume Expansion Rate	7 Days Line Expansion Rate	1 Day Compressive Strength
10~50 g/m^3^	300~400 kg/m^3^	600~700 kg/m^3^	2.5%	0.1%	6.8 MPa

**Table 2:** Properties of emulsified asphalt.

Type of Emulsified Asphalt	Solid Content	Viscosity (25 °C)	Storage Stability (1 Day, 25 °C)	Penetration (25 °C, 100 g)	Softening Point/°C
cationic	58~63%	6	0.6	68	56

**Table 3:** Mix proportions of CAM I.

Emulsified Asphalt (kg/m^3^)	Dry Materials (kg/m^3^)	Water (kg/m^3^)
550	1000	50

**Table 4:** Summary of test program.

NO.	Samples	20 °C	35 °C	45 °C	55 °C
1	fluidity of CAM I without superplasticizer	Y	Y	Y	Y
2	expansion ratio of CAM I without superplasticizer	Y	Y	Y	
3	pH value of Emulsified Asphalt-Sand system	Y			Y
4	pH value of Emulsified Asphalt-Water system	Y			Y
5	pH value of Emulsified Asphalt-Cement system	Y			Y
6	pH value of CAM I	Y	Y	Y	Y
7	fluidity of CAM I with superplasticizer			Y	
8	expansion ratio of CAM I with superplasticizer			Y	
9	IR	Y	Y	Y	Y
10	SEM	Y			Y

Note: Y refers to tested combination.
